# Triorchidism at orchidopexy: a case report

**DOI:** 10.1186/1752-1947-2-247

**Published:** 2008-07-25

**Authors:** Sharan Athwal, Jignesh Tailor, Kokila Lakhoo

**Affiliations:** 1 Department of Paediatric Surgery, John Radcliffe Hospital, Headington, Oxford, OX3 9DU, UK

## Abstract

**Introduction:**

Polyorchidism is defined as the presence of more than two testes. The management of this rare condition is still debatable, particularly when it is an incidental finding at surgery.

**Case presentation:**

We present the case of an 8-year-old boy with triorchidism found incidentally during an elective orchidopexy. This supernumerary, ectopic and atrophic testis was removed to avoid an increased risk of malignancy.

**Conclusion:**

Risk of malignancy justifies the removal of an atrophic and ectopic testis in triorchidism. However, it would appear safe to preserve a viable intrascrotal supernumerary testis found incidentally at surgery provided that the patient is followed up in the long term.

## Introduction

Polyorchidism is a rare condition defined as the presence of more than two histologically proven testes [1]. Triorchidism is the most commonly reported variety, with the supernumerary testis usually presenting on the left [2]. Its occurrence could be explained by the transverse division of the genital ridge during development and can be classified according to the extent of division (Leung's classification) [2]. Type A includes all supernumerary testes with no associated epididymis or vas deferens owing to complete division of the genital ridge. Type B includes supernumerary testes that drain into the epididymis of the normal testes. Type C includes supernumerary testes that possess their own epididymis but share a vas deferens with the regular testes. In type D polyorchidism, there is complete duplication of the testes, epididymis and vas deferens as a result of a vertical division of the genital ridge and mesonephros.

The management of supernumerary testes is still debatable, particularly when found incidentally in association with undescended testes, testicular torsion, hydrocoele or inguinal hernia. We report a rare case of triorchidism in an 8-year-old boy that was found during an elective orchidopexy and discuss current views on the management options.

## Case presentation

An 8-year-old boy presented with an undescended left testicle. Although bilateral testes were present at birth, the left testis was reported as retractile at the age of 3 years. On examination, the right testis was normal in size and well placed in the scrotum, the left testicle was palpable in the groin and the left scrotum was well developed but empty.

At operation, a normal sized testis with normal morphology was found in the left inguinal canal attached to normal testicular vessels and a vas deferens. A smaller, atrophic testis was found more distal in the suprascrotal space also attached to a normal appearing vas deferens and vessels. The two vasa deferentia arose from a single vas at the internal ring. The findings were consistent with Leung's type C polyorchidism [2]. The normal sized testis was brought down and fixed to the scrotum and the rudimentary testis was removed. Histology reports confirmed an atrophic testis.

## Discussion

Polyorchidism is a rare condition that may be found incidentally during a groin exploration. There is no consensus in the literature regarding the management of this condition, particularly when the supernumerary testis is viable, thus posing a surgical dilemma when found incidentally.

Authors proposing a conservative approach argue that infertility is a common finding in patients with polyorchidism [3] and preserving a potentially functional supernumerary testis to improve the capacity for spermatogenesis is essential even if they are found to be smaller or in ectopic locations [1]. This potential benefit is weighed against a 4% to 7% risk of malignancy in these patients [4]. With advances in radiological imaging, magnetic resonance imaging has been suggested as a safe and sensitive method for long-term surveillance of patients with polyorchidism [5]. Although this may not be cost-effective, it gives the option of preserving a functioning supernumerary testicle found incidentally if there is doubt about its long-term outcome.

Other authors argue that the majority of accessory testes have histologically reduced or absent spermatogenesis and propose that the increased risk of malignancy warrants removal of the supernumerary testis, particularly if it is non-viable, undescended or ectopically located [6,7]. This is supported by extrapolation from our knowledge of the poor fertility of simple dysplastic and ectopic testes. Singer et al. [6] proposed a functional classification based on the reproductive potential of the supernumerary testes to help in the management of triorchidism at surgery. All supernumerary testes that drain into an epididymis or vas deferens were type 1; those that are not attached to an epididymis or vas deferens were type 2. Since abnormalities of descent may have a bearing on functional outcome, these groups were further classified by the intrascrotal (type a) or ectopic (type b) location of the supernumerary testes. Singer et al. [6] recommended excision of all non-functioning (Singer type 2) or ectopically located (Singer type 1b and 2b) supernumerary testes. Excision of the supernumerary testes in type 1a triorchidism is indicated if there is at least one viable intrascrotal testis with normal drainage into a vas deferens, malignant or dysplastic change on biopsy, absent spermatogenesis, an ultrasound scan suggestive of malignancy, a desire by the patient to have a single testis in each hemiscrotum or circumstances where regular follow-up is unlikely to be reliable [6]. In this case the decision to remove the supernumerary testis was less challenging owing to the atrophic nature of the testis. Interestingly, it is unusual to find the smaller testes distal to the larger testes [8], as was found in our case. Since the increased risk of malignancy in dysplastic, undescended or ectopic testes is well known [6], it would appear reasonable to remove an ectopically located accessory testis. With current advances in imaging, and patient self-examination, it would appear safe to preserve a viable intrascrotal supernumerary testis found incidentally provided that the patient receives appropriate long-term follow-up.

## Conclusion

This report illustrates the unexpected presentation of polyorchidism during elective groin procedures and the management challenge considering the risk and benefits of excising a supernumerary viable testis.

## Competing interests

The authors declare that they have no competing interests.

## Authors' contributions

SA presented the case history, researched the topic and helped draft the manuscript. JT reviewed the literature and drafted the manuscript. KL was the supervising consultant who performed the surgery, obtained consent for publication and edited the manuscript. All authors read and approved the final manuscript.

## Consent

Written informed consent was obtained from the patient's next-of-kin for publication of this case report. A copy of the written consent is available for review by the Editor-in-Chief of this journal.
